# The Italian Version of the Drooling Impact Scale: Translation and Psychometric Validation in Children with Neurodevelopmental Conditions

**DOI:** 10.3390/children13060757

**Published:** 2026-05-29

**Authors:** Federica Pauciulo, Marco Tofani, Giulia Stella, Alessandra Lacopo, Susanna Summa, Giulia Tullo, Caterina Delia, Antonella Cerchiari, Gessica Della Bella

**Affiliations:** 1 Management and Diagnostic Innovations & Clinical Pathways Research Area, Neurorehabilitation and Adapted Physical Activity Day Hospital, Bambino Gesù Children’s Hospital, IRCCS, 00165 Rome, Italy; federicapauciulo@gmail.com (F.P.); marco.tofani@opbg.net (M.T.); giulia.stella@opbg.net (G.S.); alessandra.lacopo@opbg.net (A.L.); susanna.summa@opbg.net (S.S.); giulia.tullo@gmail.com (G.T.); caterina.delia@opbg.net (C.D.); antonella.cerchiari@opbg.net (A.C.); 2 Department of Life Sciences, Health and Healthcare Professions, Università degli Studi “Link Campus University”, 00165 Rome, Italy; 3 Pediatric Neurology Unit, Catholic University of Sacred Heart, 00168 Rome, Italy

**Keywords:** drooling, neurodevelopmental disorders, pediatric rehabilitation, psychometric validation, patient-reported outcome measures, DIS, measurement properties, COSMIN

## Abstract

**Highlights:**

**What are the main findings?**

- The Italian version of the Drooling Impact Scale shows excellent internal consistency and strong inter-rater and test–retest reliability in children with neurodevelopmental conditions.
- Structural validity supports a predominantly unidimensional construct, with a single factor explaining most of the variance.

**What are the implications of the main findings?**

- The Italian DIS can be used as a reliable caregiver-reported outcome measure to assess the impact of drooling in both clinical and research settings.
- Measurement error estimates support its use in group-level studies, while interpretation at the individual level requires caution when evaluating change over time.

**Abstract:**

Background/Objectives: Drooling is a common and clinically relevant issue in children with neurodevelopmental conditions, with important consequences for daily functioning, social participation, and caregiver burden. The lack of validated tools in Italian makes it difficult to quantify the impact of drooling on daily life, support appropriate care pathways, and evaluate the effectiveness of interventions. The aim of this study was to translate, culturally adapt, and evaluate the psychometric properties of the Italian version of the Drooling Impact Scale (DIS) in a pediatric population. Methods: The DIS is a 10-item caregiver-reported outcome measure, with each item rated on an ordinal 0–10 scale, designed to assess the functional and psychosocial impact of drooling. It was translated using a standard forward–backward procedure, followed by expert review and cognitive debriefing with caregivers. Caregivers of children aged ≥2 years with heterogeneous neurodevelopmental conditions and feeding/swallowing impairments were consecutively recruited from a tertiary pediatric hospital. Psychometric properties were assessed in line with COSMIN recommendations, including internal consistency (Cronbach’s α), structural validity through exploratory factor analysis, inter-rater and test–retest reliability (intraclass correlation coefficients, ICC), measurement error (standard error of measurement, SEM; smallest detectable change, SDC), and construct validity through correlation with the Pediatric Quality of Life Inventory (PedsQL). Results: The Italian DIS was completed by caregivers of 126 children. It showed excellent internal consistency (Cronbach’s α = 0.92). Factor analysis indicated a clear dominant factor, explaining 56.5% of the variance, while additional factors contributed only marginally. Agreement between caregivers was excellent (ICC = 0.94), and test–retest reliability was good (ICC = 0.85). Measurement error analysis yielded SEM = 8.66, SDC_individual = 24.00, and SDC_group = 2.14. As expected, DIS scores were associated with health-related quality of life. Conclusions: The Italian version of the DIS appears to be a reliable and structurally sound instrument for assessing the impact of drooling in children with neurodevelopmental conditions. It may be useful in both clinical practice and research, although further studies are needed to explore its responsiveness and confirm these findings in different settings.

## 1. Introduction

Drooling, defined as excessive salivation, is commonly observed during infancy and is generally considered a physiological phenomenon. It typically resolves by 15 to 18 months of age as oral motor control matures. When drooling persists beyond early childhood, particularly after the age of 4 years, it is usually regarded as a pathological condition [1]. The prevalence of chronic drooling is estimated at around 60% among children with Cerebral Palsy (CP) [2] and less frequently in neurodevelopmental conditions and in rare syndromes [3,4].

Clinically, two main forms of drooling are distinguished: anterior drooling, characterized by the unintentional loss of saliva from the oral cavity, and posterior drooling, which refers to the pooling and posterior spillage of saliva into the oropharynx [5]. While anterior drooling is readily observable and primarily associated with functional and hygienic challenges, posterior drooling is often less evident and may remain under-recognized in clinical practice. Importantly, posterior drooling is associated with a higher risk of aspiration, as saliva may enter the lower airways, potentially leading to recurrent respiratory infections, chronic lung disease, and respiratory morbidity [6,7]. In severe cases, chronic aspiration of saliva can contribute to long-term pulmonary complications and increased healthcare burden.

Both forms of drooling have a substantial impact on the daily life of affected children and their caregivers [8]. From a functional perspective, drooling may interfere with feeding, communication, and oral motor performance, while also increasing the risk of skin irritation and breakdown due to constant moisture exposure [9]. Hygienic challenges, such as the need for frequent clothing changes and continuous wiping of saliva, can significantly increase caregiver burden. Moreover, the psychosocial impact of drooling is considerable [10]. Children with visible drooling may experience social stigma, reduced participation in social activities, and difficulties in peer interactions, which can negatively affect self-esteem and overall well-being [11].

The burden extends beyond the child to the family, as caregivers often report increased stress, emotional strain, and limitations in daily activities related to the management of drooling [8,9,10]. Taken together, these findings highlight that drooling is not merely a physical symptom but a multifaceted condition with significant functional, medical, and psychosocial consequences, underscoring the importance of comprehensive assessment and management strategies.

A recent systematic review [12] highlighted the limited availability of validated outcome measures specifically designed to assess drooling in children with neurodevelopmental conditions. Existing instruments differ substantially in their aims and constructs. Some tools, such as the 5 min Drooling Quotient [13], primarily focus on the objective quantification of drooling frequency and severity, whereas the Drooling Impact Scale (DIS) [14] was specifically developed to assess the functional, psychosocial, and caregiver-related impact of drooling, as well as changes following treatment. Considering the importance of multidisciplinary management of drooling and the need for outcome measures able to capture clinically meaningful changes in daily life, the present study aimed to translate, culturally adapt, and validate the DIS for the Italian population of children with neurodevelopmental conditions. In line with these aims, a set of a priori hypotheses was defined according to COSMIN recommendations [15,16]. The Italian version of the DIS was expected to demonstrate high internal consistency and good reliability, both between caregivers and over time. Based on findings from previous validation studies, a bidimensional structure was anticipated. Finally, an association between DIS scores and health-related quality of life was expected.

## 2. Materials and Methods

The present cross-sectional study has been designed to evaluate the psychometric properties of the DIS in a pediatric Italian population with neurodevelopmental conditions. The study was approved by the Ethics Committee of Bambino Gesù Children’s Hospital with protocol number 3329_OPBG_2024.

### 2.1. Assessment Tools

The Drooling Impact Scale (DIS) is a caregiver-reported outcome measure specifically developed to assess the functional and psychosocial impact of drooling in children with developmental and neurological conditions. It was originally introduced by Reid and colleagues [14], and has been translated into French [17], Brazilian Portuguese [18] and Arabic [19]. DIS consists of 10 items, each addressing different domains related to drooling, including daily management (e.g., need for clothing changes), physical consequences (e.g., skin irritation), and social or emotional impact (e.g., embarrassment or limitations in participation). The DIS consists of 10 items, each scored on a fixed ordinal scale ranging from 0 to 10. The total score is obtained by summing all items, resulting in a range from 0 to 100, where higher values reflect a more severe impact on the child and caregiver. From a psychometric perspective, the DIS has demonstrated good measurement properties across studies [14,17,18,19].

The Pediatric Quality of Life Inventory (PedsQL) is a widely used generic instrument designed to assess health-related quality of life in children and adolescents [20]. It was developed to provide a standardized, multidimensional evaluation of physical, emotional, social, and school functioning, and can be applied across both healthy and clinical populations. The most commonly used version, the PedsQL 4.0 Generic Core Scales [21], includes 23 items organized into four domains: Physical Functioning (8 items), Emotional Functioning (5 items), Social Functioning (5 items), and School Functioning (5 items). Both child self-report (when developmentally appropriate) and parent proxy-report versions are available, allowing assessment across a wide age range and clinical contexts. Items are rated on a 5-point Likert scale (0 = never a problem to 4 = almost always a problem). Scores are reverse-scored and linearly transformed to a 0–100 scale, with higher scores indicating better health-related quality of life. For the present investigation, the Total Score has been used.

### 2.2. Translation

The translation and cross-cultural adaptation of the DIS were carried out in accordance with established international recommendations [22] for patient-reported outcome measures. First, we asked for formal permission from the original developer of the DIS. The process started with two independent forward translations from the original language into Italian, performed by bilingual translators with Italian as their native language. One translator had a clinical background and was familiar with the construct of interest, while the other had no specific knowledge of the instrument, to capture both conceptual accuracy and natural language use. The two versions were compared and merged into a single reconciled version through discussion within the research team.

The reconciled Italian version was then translated back into the original language by two independent translators who were native English speakers and blinded to the original instrument. The backward translations were compared with the original version to identify discrepancies in meaning, wording, or conceptual equivalence. An expert panel, including Physical and Rehabilitation Medicine doctors, speech and language therapists, and occupational therapists, reviewed all versions to ensure semantic and conceptual consistency. This process resulted in a pre-final Italian version of the DIS.

The pre-final version was subsequently tested through cognitive debriefing with 8 caregivers of children with neurodevelopmental conditions recruited from the same clinical setting. Caregivers were asked to complete the questionnaire and participate in a brief semi-structured discussion focused on item clarity, comprehensibility, wording, and interpretation of response options. Feedback was collected directly by the research team and analyzed qualitatively to identify linguistic or conceptual difficulties. Modifications to the wording were introduced when recurrent comprehension issues or culturally inappropriate expressions were identified across participants.

Following this step, and after approval from the original developer of the DIS, the final Italian version was established, ensuring that the instrument retained the meaning of the original items while being fully understandable and appropriate within the Italian cultural context (Please see Supplementary Material S1).

### 2.3. Sampling

The sample included children aged ≥2 years with neurodevelopmental conditions associated with feeding and/or swallowing impairments and clinically relevant drooling. Participants were consecutively recruited from specialized pediatric neurorehabilitation services at Bambino Gesù Children’s Hospital. Diagnoses were established by the treating multidisciplinary clinical team, including Physical and Rehabilitation Medicine physicians, Speech and Language Therapists, and Occupational Therapists, based on standard clinical assessment and medical records. The presence of clinically relevant drooling was identified through routine multidisciplinary clinical evaluation and caregiver report during the rehabilitation assessment process. Inclusion criteria also require the availability of a primary caregiver able to complete the questionnaire. Children with acute or unstable medical conditions, or with clinical situations that could compromise the reliability of the assessment, were not included.

Sample size considerations were guided by recommendations for studies on measurement properties. A subject-to-item ratio of 5–10 participants per item is generally considered sufficient for exploratory factor analysis. Given the 10-item structure of the DIS, this would correspond to a minimum of 50 to 100 participants. However, to enhance the stability of the factor solution, the study aimed to include at least 100 participants, in line with COSMIN recommendations for structural validity. The final sample met this target, supporting the robustness of the analyses conducted.

### 2.4. Data Analysis

The psychometric properties of the DIS were examined through a comprehensive set of analyses addressing internal consistency, structural validity, reliability, measurement errors, and construct validity, in accordance with established methodological standards for PROMs.

Internal consistency [23] was evaluated using Cronbach’s alpha coefficient to assess the degree of interrelatedness among items, with values ≥0.70 considered indicative of acceptable homogeneity and higher thresholds reflecting increasing levels of reliability [24]. Item-total correlations and the impact of item removal on the overall alpha coefficient were also inspected to ensure that each item contributed meaningfully to the underlying construct. Floor and ceiling effects were examined at both item and total-score levels. Effects exceeding 15% were considered potentially relevant.

Structural validity was assessed through exploratory factor analysis (EFA) [25] using the principal axis factoring method. Since DIS items are scored on an ordinal 0–10 response scale, the variables were treated as approximately continuous, consistent with common psychometric practice for scales with a relatively large number of response categories. Accordingly, factor analysis was performed using the standard correlation matrix. Prior to extraction, the suitability of the data for factor analysis was examined by inspecting the correlation matrix and calculating the Kaiser–Meyer–Olkin measure of sampling adequacy [26], together with Bartlett’s test of sphericity [27]. Factors were initially extracted based on the Kaiser criterion (eigenvalues greater than one) and further evaluated through visual inspection of the scree plot. To strengthen factor retention decisions, parallel analysis was also performed. Parallel analysis compares the observed eigenvalues obtained from the study data with eigenvalues generated from random datasets with the same number of observations and variables. Factors were retained only when the observed eigenvalue exceeded the corresponding randomly generated eigenvalue. This approach was used to complement the Kaiser criterion and scree plot inspection, reducing the risk of over-extracting factors. No a priori restriction on the number of factors was imposed. The factor solution was initially examined without rotation; where appropriate, rotation was considered to improve interpretability [28]. Factor loadings and communalities were inspected to evaluate the contribution of each item to the latent structure, with factor loadings ≥0.30 considered indicative of meaningful association [29].

Reliability was further examined through both inter-rater and intra-rater analyses [30]. Inter-rater reliability, specifically between parental respondents (parent 1 and parent 2), was assessed using the intraclass correlation coefficient (ICC) derived from a two-way random-effects single-measure model with absolute agreement (ICC [2,1]). Caregivers completed the questionnaires independently from one another. Intra-rater reliability (test–retest reliability) was evaluated using the same ICC model (ICC [2,1]), by comparing scores obtained at two time points (T0 and T1) separated by a 14-day interval and completed by the same caregiver. During this interval, no relevant clinical changes were expected or reported. ICC values were interpreted according to conventional thresholds, with values above 0.70 indicating good reliability and values exceeding 0.90 reflecting excellent agreement [31].

Measurement error was quantified by calculating the standard error of measurement (SEM) and the smallest detectable change (SDC). SEM was calculated using the standard deviation of the test–retest sample and the ICC [2,1] reliability coefficient according to the formula SEM = SD × √(1 − ICC). The SDC at the individual level was calculated as SDC_individual = 1.96 × √2 × SEM, while the SDC at the group level, SDC_group = SDC_individual/√n), where n represents the number of participants included in the test–retest analysis. These indices allow the interpretation of change both at the individual and group levels, depending on the intended application of the instrument.

Construct validity [32] was assessed by examining the association between DIS scores and health-related quality of life, as measured by the parent-proxy version of the Pediatric Quality of Life Inventory (PedsQL). Age-appropriate Italian versions of the PedsQL were administered according to the child’s age. For children not attending school, school functioning items were treated according to the official PedsQL scoring guidelines. Given the distributional characteristics of the data, Spearman’s rank correlation coefficient was used to evaluate the strength and direction of the relationship between the two measures [33]. A priori, we hypothesized a moderate negative correlation between DIS and PedsQL total scores (ρ ≥ 0.40), assuming that higher drooling impact would be associated with lower health-related quality of life. No relevant missing data were observed.

## 3. Results

A total of 126 children were recruited. The mean age of participants was 9.6 years (SD 4.3), and the sample included 58% males and 42% females. The characteristics of the sample are reported in Table 1 and Table 2.

No relevant floor or ceiling effects were observed for the DIS total score. At the item level, floor effects were generally low, whereas ceiling effects exceeding 15% were observed for some items, particularly items 1 (22.8%), 3 (22.8%), and 6 (24.6%). Internal consistency, as measured by Cronbach’s alpha, showed a value of 0.92. Table 3 reports item-total statistics of the DIS.

Structural validity was examined using exploratory factor analysis with principal axis factoring. The correlation matrix showed several moderate-to-high inter-item correlations, supporting the assumption of an underlying common construct. Sampling adequacy was confirmed by a Kaiser–Meyer–Olkin value of 0.87, and Bartlett’s test of sphericity was significant (χ^2^ = 856.61, df = 45, *p* < 0.001), indicating that the data were suitable for factor analysis. Initial extraction identified two factors with eigenvalues greater than one. The eigenvalues of the first and second factors were 5.96 and 1.00, respectively. The first factor accounted for 56.5% of the variance, whereas the second factor explained 5.5%. Inspection of the scree plot showed a clear inflection after the first factor. Parallel analysis indicated that only the first observed eigenvalue exceeded the corresponding randomly generated eigenvalue (1.64), whereas the second observed eigenvalue did not exceed the corresponding random eigenvalue (1.42). Communalities ranged from 0.25 to 0.81. Table 4 reports factor loadings and communalities for the EFA of the DIS.

Regarding reliability analysis, as measured by ICC, our findings reported values of 0.94 and 0.85 for inter-rater and test–retest reliability, respectively. Mean, SD and ICC 95% CI for both inter-rater and test–retest reliability are reported in Table 5. The inter-rater (n = 32) and test–retest (n = 48) subsamples showed demographic and clinical characteristics comparable to those of the full sample.

Measurement error analysis showed an SEM of 8.66. The SDC at the individual level (SDC_individual) was 24.00, indicating the magnitude of change required to be confident that a real change has occurred beyond measurement error for a single subject. At the group level, the SDC_group was considerably lower (2.14), supporting the use of the instrument for detecting changes in research and group comparisons. These findings provide information on the precision of DIS scores and their interpretability in both clinical and research contexts.

Spearman’s rank correlation analysis was performed to examine the association between DIS scores (median 49, IQR 29–69) and PedsQL total scores (median 33, IQR 27–39). A moderate negative association between DIS total scores and PedsQL total scores (ρ = −0.52, *p* < 0.001) was observed, consistent with the a priori hypothesis that greater drooling impact would be associated with lower health-related quality of life.

## 4. Discussion

The present study provides a comprehensive evaluation of the Italian version of the Drooling Impact Scale (DIS), demonstrating overall solid psychometric properties. In particular, the instrument showed excellent internal consistency (Cronbach’s α = 0.92), high inter-rater reliability between caregivers (ICC = 0.94), and good test–retest reliability over time (ICC = 0.85), alongside acceptable levels of measurement error (SEM = 8.66; SDC_individual = 24.00). Furthermore, structural validity analyses supported a predominantly unidimensional structure, with exploratory factor analysis identifying a dominant first factor explaining 56.5% of the variance, while additional factors contributed only marginally. These findings collectively indicate that the Italian DIS is a reliable and structurally coherent tool for assessing the impact of drooling in pediatric populations with feeding and swallowing impairments.

The translation and cross-cultural adaptation process represented a critical phase of the study and substantially contributed to the overall quality of the instrument [34]. In line with established methodological standards, the process combined forward–backward translation, expert review, and cognitive debriefing. The pre-test phase proved particularly important in identifying linguistic expressions that, although technically accurate, were perceived as unclear or culturally inappropriate by caregivers. For example, the medical term “scialorrea,” initially adopted as a direct translation of “drooling,” was poorly understood by several caregivers. Similarly, the literal translation “sbavare” was perceived as excessively colloquial and potentially negatively connoted in the context of children with neurodevelopmental conditions. Based on caregiver feedback, the more neutral and comprehensible expression “perdita di saliva” (“saliva loss”) was consistently adopted throughout the questionnaire, including in items referring to drooling or salivation. These modifications aimed to improve clarity, reduce potentially stigmatizing language, and ensure conceptual and terminological consistency while preserving the meaning of the original instrument. This finding highlights the importance of prioritizing conceptual clarity and respondent understanding over strict literal translation, particularly in caregiver-reported measures. Similar issues have been documented in other cross-cultural adaptation studies, reinforcing the value of iterative pre-testing in achieving semantic and cultural equivalence.

With regard to structural validity, the findings of the present study support a predominantly unidimensional interpretation of the DIS. Although the Kaiser criterion initially identified two factors with eigenvalues greater than one, several complementary findings supported the retention of a dominant single-factor solution. The first factor accounted for a substantial proportion of variance (56.5%), whereas the second factor explained only a limited additional proportion (5.5%). Furthermore, inspection of the scree plot showed a clear inflection after the first factor, and parallel analysis supported the retention of only one factor, as the second observed eigenvalue did not exceed the corresponding randomly generated eigenvalue. In addition, most items loaded strongly on the first factor, while loadings on the second factor did not reveal a clinically or theoretically coherent pattern. This result contrasts with previous adaptations, such as the Brazilian Portuguese [18] and Saudi [19] versions, which reported a two-factor structure. However, these findings should be interpreted in light of methodological considerations, particularly sample size. The Brazilian Portuguese validation included approximately 40 participants, while the Saudi study involved 72 children, both below the thresholds generally recommended by COSMIN for robust structural validity analyses. In contrast, the present study deliberately targeted a sample size of at least 100 participants, thereby ensuring greater stability of the factor solution and more reliable estimation of latent structure. This difference in sample adequacy may partially explain the discrepancy in dimensional findings across studies. Nevertheless, some items (particularly items 4 and 7) showed lower communalities and weaker loadings compared with the remaining items, suggesting that specific aspects of drooling impact, especially those related to social or contextual dimensions, may be less strongly represented by the general latent construct. Future studies using larger independent samples and confirmatory approaches may help further clarify the dimensional structure of the DIS across different cultural contexts.

The internal consistency observed in this study (α = 0.92) indicates a high degree of interrelatedness among items, suggesting that they measure a common underlying construct. This value is higher than those reported in several previous adaptations, including the Brazilian Portuguese (α = 0.72) [18] and French (α = 0.71) [17] versions, and comparable to the original (0.85) [14] and Saudi version (α = 0.91) [19]. While high alpha values may sometimes raise concerns about item redundancy, the inspection of item-total correlations and alpha-if-deleted statistics suggests that each item contributes meaningfully to the overall scale. Nonetheless, the slightly lower corrected item-total correlations and communalities observed for specific items (notably items 4 and 7) indicate that these items may capture more peripheral or context-dependent aspects of drooling impact, such as social perception or environmental factors, rather than the core physical dimension.

Reliability analyses further support the robustness of the instrument. The inter-rater reliability between caregivers (ICC = 0.94) indicates excellent agreement, suggesting that the instrument provides stable estimates of the construct regardless of the respondent, provided they are familiar with the child’s condition. Similarly, the test–retest reliability (ICC = 0.85) confirms the temporal stability of the measure in conditions where no clinical change is expected. These findings are consistent with those reported in other versions, such as the French adaptation (ICC = 0.83) [17], reinforcing the reproducibility of the DIS across different cultural contexts.

Measurement error indices provide additional insight into the interpretability of DIS scores. The SEM value (8.66) reflects the expected variability in repeated measurements, while the SDC values indicate the minimum change required to be confident that a real change has occurred beyond measurement error. In particular, the relatively large individual-level SDC (24.00) suggests that substantial changes in total score are required to be confident that observed score differences exceed measurement error at the individual level, whereas the much smaller group-level SDC (2.14) supports the use of the instrument in research contexts and group comparisons. These findings are aligned with COSMIN recommendations, emphasizing the importance of distinguishing between individual and group-level interpretation when considering score changes over time. However, SDC values should not be interpreted as indicators of clinical meaningfulness or responsiveness, since minimal important change and responsiveness were not evaluated in the present study.

Overall, the results of this study are consistent with, and in some aspects extend, the existing literature on the DIS [12]. The combination of a sufficiently large sample, rigorous methodological approach, and comprehensive evaluation of measurement properties strengthens the evidence supporting the use of the Italian version of the DIS. Importantly, the findings suggest that the scale primarily captures a single underlying construct related to the impact of drooling, while still incorporating items that reflect broader psychosocial and contextual dimensions. This balance enhances the clinical relevance of the instrument, allowing for a multidimensional yet coherent assessment of drooling in children with complex health conditions.

### Limitations

This study has several limitations that should be considered when interpreting the findings. First, responsiveness was not evaluated. Although the present study provides robust evidence on reliability and measurement error, the ability of the DIS to detect clinically meaningful changes over time remains unknown. This property is particularly relevant in the context of clinical trials and longitudinal interventions, where outcome measures are expected to capture change following treatment. Future studies should therefore investigate responsiveness, ideally integrating anchor-based and distribution-based approaches. When combined with the measurement error estimates reported in the present study (SEM and SDC), such analyses would substantially strengthen the interpretability of score changes and enhance the applicability of the instrument in both clinical practice and research settings. Second, although the sample size was adequate according to COSMIN recommendations for structural validity, the study was conducted in a single tertiary care center. This may limit the external validity and generalizability of the findings, particularly to community settings or to populations with different socio-demographic or clinical characteristics. Multicenter studies would be beneficial to confirm the stability of the psychometric properties across contexts. Furthermore, although inter-rater reliability between caregivers was assessed and found to be excellent, the number of paired observations (n 32) was relatively limited. This may affect the precision of the ICC estimates and warrants confirmation in larger samples.

## 5. Conclusions

The Italian version of the DIS demonstrated good overall psychometric properties in a pediatric population with neurodevelopmental conditions, including high internal consistency, strong inter-rater agreement, and good test–retest reliability. Structural validity analyses supported a predominantly unidimensional construct, with a single factor accounting for the majority of the variance. Measurement error estimates indicated acceptable precision at the group level, while suggesting caution when interpreting changes at the individual level. Overall, the instrument appears suitable for use in clinical research and for cross-sectional assessment of the impact of drooling in clinical practice. However, caution is warranted when interpreting score changes over time at the individual level, as responsiveness and minimal important change were not evaluated in the present study. Further longitudinal studies are warranted to evaluate responsiveness, minimal important change, and the applicability of the instrument for individual treatment monitoring over time.

## Figures and Tables

**Table 1:** Sample characteristics (total 126).

**Variable**	**Value**
Age, mean (SD), years	9.6 (4.3)
Gender *n* (%) M/F	73 (58)/53 (42)
**Diagnoses**	***n* (%)**
Cerebral palsy	50 (39.7%)
Genetic syndromes	44 (34.9%)
Monogenic disorders	25 (19.8%)
Other neurological conditions	7 (5.6%)
**School Attendance**	***n* (%)**
Pre-school (nursery/kindergarten)	48 (38.1%)
Primary school	39 (31.0%)
Secondary school	23 (18.3%)
Not attending school	9 (7.1%)
Other/unspecified	7 (5.6%)

**Table 2:** Score distribution of the DIS.

Item	DIS	Mean (SD)	Median (Q25–Q75)
1	How frequently did your child dribble?	7.11 (2.18)	8 (6.5–9.5)
2	How severe was the drooling?	6.84 (2.27)	7 (5–9)
3	How many times a day did you have to change bibs or clothing due to drooling?	5.95 (3.01)	5 (2–8)
4	How offensive was the smell of the saliva on your child?	2.58 (2.24)	2 (0.5–3.5)
5	How much skin irritation has your child had due to drooling?	3.26 (2.33)	3 (1–5)
6	How frequently did your child’s mouth need wiping?	6.65 (2.79)	7 (4.5–8.5)
7	How embarrassed did your child seem to be about his/her dribbling?	2.26 (2.54)	1 (0–2)
8	How much do you have to wipe or clean saliva from household items, e.g., toys, furniture, computers?	5.42 (3.24)	5 (2–8)
9	To what extent did your child’s drooling affect his or her life?	5.26 (3.49)	5 (1.5–8.5)
10	To what extent did your child’s dribbling affect you and your family’s life?	5.79 (3.54)	5 (1–9)
	Total	51.74 (20.00)	49 (29–69)

**Table 3:** Item-total statistics of the Italian version of the DIS.

Item-Total Statistics					
	Scale Mean If Item Deleted	Scale Variance If Item Deleted	Corrected Item-Total Correlation	Squared Multiple Correlation	Cronbach’s Alpha If Item Deleted
ITEM 1	44.11	406.785	0.811	0.878	0.908
ITEM 2	44.51	402.270	0.848	0.892	0.906
ITEM 3	45.28	384.770	0.837	0.744	0.905
ITEM 4	48.05	446.422	0.520	0.408	0.922
ITEM 5	47.56	424.532	0.604	0.440	0.918
ITEM 6	44.46	405.631	0.784	0.719	0.909
ITEM 7	48.77	451.576	0.436	0.341	0.926
ITEM 8	45.75	397.355	0.729	0.626	0.912
ITEM 9	46.19	386.316	0.762	0.683	0.910
ITEM 10	45.26	393.045	0.703	0.641	0.914

**Table 4:** Exploratory Factor Analysis of the DIS.

Item	Factor 1	Factor 2	Communality
Item 1	0.87	−0.28	0.76
Item 2	0.91	−0.28	0.81
Item 3	0.87	0.01	0.78
Item 4	0.53	0.15	0.34
Item 5	0.62	0.03	0.46
Item 6	0.83	−0.16	0.72
Item 7	0.45	0.32	0.25
Item 8	0.77	−0.16	0.64
Item 9	0.80	0.41	0.66
Item 10	0.73	0.24	0.68

**Table 5:** Inter-rater and test–retest reliability of the DIS.

Item Statistics			
**Inter-Rater Reliability (Tot 32)**			
**DIS**	**Mean (SD)**	**Median (Q25–Q75)**	**ICC 95% CI**
Parent 1	51.74 (20.0)	49 (29–69)	0.94 (0.88–0.97)
Parent 2	50.1 (19.8)	48 (31–66)	
**Test-Retest Reliability (Tot 48)**			
**DIS**	**Mean (SD)**	**Median (Q25–Q75)**	**ICC 95% CI**
Time 1	49.7 (18.8)	48 (31–65)	0.85 (0.75–0.92)
Time 2	48.7 (20.7)	48 (30–66)	

## Data Availability

Data are available from the corresponding author upon reasonable request. The data are not publicly available due to institutional and ethical restrictions related to informed consent procedures and the protection of participant privacy.

## Supplementary Materials

The following supporting information can be downloaded at: https://www.mdpi.com/article/10.3390/children13060757/s1, File S1: Translation process of the Italian version of the DIS.

## References

[1] Paine C.C., Snider J.W. (2020). When Saliva Becomes a Problem: The Challenges and Palliative Care for Patients with Sialorrhea. Ann. Palliat. Med..

[2] Speyer R., Cordier R., Kim J., Cocks N., Michou E., Wilkes-Gillan S. (2019). Prevalence of Drooling, Swallowing, and Feeding Problems in Cerebral Palsy across the Lifespan: A Systematic Review and Meta-analyses. Dev. Med. Child Neurol..

[3] Riva A., Federici C., Piccolo G., Amadori E., Verrotti A., Striano P. (2021). Exploring Treatments for Drooling in Children with Neurological Disorders. Expert Rev. Neurother..

[4] Riva A., Amadori E., Vari M.S., Spalice A., Belcastro V., Viri M., Capodiferro D., Romeo A., Verrotti A., Aiello M.F. (2022). Impact and Management of Drooling in Children with Neurological Disorders: An Italian Delphi Consensus. Ital. J. Pediatr..

[5] Delsing C.P., Bekkers S., Erasmus C.E., van Hulst K., van den Hoogen F.J. (2021). Posterior Drooling in Children with Cerebral Palsy and Other Neurodevelopmental Disorders. Dev. Med. Child Neurol..

[6] Reddihough D.S., Chang A.B. (2021). The Challenges of Posterior Drooling in Children with Cerebral Palsy. Dev. Med. Child Neurol..

[7] Speyer R. (2013). Oropharyngeal Dysphagia. Otolaryngol. Clin. N. Am..

[8] Orriëns L.B., van der Burg J.J.W., Maille-Wisse P., de Boer A., van den Hoogen F.J.A., Willemsen M.A.A.P., van Hulst K., Erasmus C.E. (2025). Understanding Posterior Drooling in Children and Young People with Neurodevelopmental Disabilities: A Parental Perspective. BMC Pediatr..

[9] Chang S.C., Lin C.K., Tung L.C., Chang N.Y. (2012). The Association of Drooling and Health-Related Quality of Life in Children with Cerebral Palsy. Neuropsychiatr. Dis. Treat..

[10] Gandhi K., Strychowsky J.E., Chen B.A. (2024). Pediatric Sialorrhea (Drooling). Can. Med. Assoc. J..

[11] Ashraf M., Ashraf A., Imtiaz R., Azmat R. (2023). Effect of Sialorrhea on Quality of Life in Cerebral Palsy Children. J. Health Rehabil. Res..

[12] Sforza E., Onesimo R., Leoni C., Giorgio V., Proli F., Notaro F., Kuczynska E.M., Cerchiari A., Selicorni A., Rigante D. (2022). Drooling Outcome Measures in Paediatric Disability: A Systematic Review. Eur. J. Pediatr..

[13] van Hulst K., Lindeboom R., van der Burg J., Jongerius P. (2012). Accurate Assessment of Drooling Severity with the 5-min Drooling Quotient in Children with Developmental Disabilities. Dev. Med. Child Neurol..

[14] Reid S.M., Johnson H.M., Reddihough D.S. (2010). The Drooling Impact Scale: A Measure of the Impact of Drooling in Children with Developmental Disabilities. Dev. Med. Child Neurol..

[15] Mokkink L.B., Boers M., van der Vleuten C.P.M., Bouter L.M., Alonso J., Patrick D.L., de Vet H.C.W., Terwee C.B. (2020). COSMIN Risk of Bias Tool to Assess the Quality of Studies on Reliability or Measurement Error of Outcome Measurement Instruments: A Delphi Study. BMC Med. Res. Methodol..

[16] Terwee C.B., Prinsen C.A.C., Chiarotto A., Westerman M.J., Patrick D.L., Alonso J., Bouter L.M., de Vet H.C.W., Mokkink L.B. (2018). COSMIN Methodology for Evaluating the Content Validity of Patient-Reported Outcome Measures: A Delphi Study. Qual. Life Res..

[17] Bard-Pondarré R., Roumenoff F., Julien C., Grguric G., Porte M., Boulay C., Bourg V., Chaléat-Valayer E. (2022). Validity, Reliability and Responsiveness to Change of the French Version of the Drooling Impact Scale. Disabil. Rehabil..

[18] Cavalcanti N.S., Sekine L., Manica D., Farenzena M., Saleh Neto C.D.S., Marostica P.J.C., Schweiger C. (2022). Translation and Validation of the Drooling Impact Scale Questionnaire into Brazilian Portuguese. Braz. J. Otorhinolaryngol..

[19] Alturki L.S., Almutairi N.K., Alghamdi S.M., Allami H.A., Alhawamdeh S.T., AlMakoshi L.A. (2025). Arabic Translation and Validation of the Drooling Impact Scale Questionnaire. Saudi J. Otorhinolaryngol. Head Neck Surg..

[20] Varni J.W., Burwinkle T.M., Seid M. (2005). The PedsQL^TM^ as a Pediatric Patient-Reported Outcome: Reliability and Validity of the PedsQL^TM^ Measurement Model in 25,000 Children. Expert Rev. Pharmacoecon. Outcomes Res..

[21] Pedrinelli I., Biagi S., Romeo D.M., Musto E., Fagiani V., Lanza M., Guastafierro E., Colombo A., Giordano A., Montomoli C. (2025). Construct Validity and Internal Consistency of the Italian Version of the PedsQLTM 4.0 Generic Core Scale and PedsQLTM 3.0 Cerebral Palsy Module. Children.

[22] Beaton D.E., Bombardier C., Guillemin F., Ferraz M.B. (2000). Guidelines for the Process of Cross-Cultural Adaptation of Self-Report Measures. Spine.

[23] Tang W., Cui Y., Babenko O. (2014). Internal Consistency: Do We Really Know What It Is and How to Assess It?. J. Psychol. Behav. Sci..

[24] Green S.B., Yang Y. (2015). Evaluation of Dimensionality in the Assessment of Internal Consistency Reliability: Coefficient Alpha and Omega Coefficients. Educ. Meas. Issues Pract..

[25] Fabrigar L.R., MacCallum R.C., Wegener D.T., Strahan E.J. (1999). Evaluating the Use of Exploratory Factor Analysis in Psychological Research. Psychol. Methods.

[26] Cerny B.A., Kaiser H.F. (1977). A Study of A Measure of Sampling Adequacy for Factor-Analytic Correlation Matrices. Multivar. Behav. Res..

[27] Bartlett M.S. (1951). The Effect of Standardization on a χ^2^ Approximation in Factor Analysis. Biometrika.

[28] Costello A.B., Osborne J. (2005). Best Practices in Exploratory Factor Analysis: Four Recommendations for Getting the Most from Your Analysis. Res. Eval. Pract. Assess. Res. Eval..

[29] Tavakol M., Wetzel A. (2020). Factor Analysis: A Means for Theory and Instrument Development in Support of Construct Validity. Int. J. Med. Educ..

[30] Shrout P.E., Fleiss J.L. (1979). Intraclass Correlations: Uses in Assessing Rater Reliability. Psychol. Bull..

[31] Nunnally J.C. (1979). Psychometric Theory.

[32] Lakshmi S., Akbar Mohideen M. (2013). Issues in reliability and validity of research. Int. J. Manag. Res. Rev..

[33] Sedgwick P. (2014). Spearman’s Rank Correlation Coefficient. BMJ.

[34] Cruchinho P., López-Franco M.D., Capelas M.L., Almeida S., Bennett P.M., Miranda da Silva M., Teixeira G., Nunes E., Lucas P., Gaspar F. (2024). Translation, Cross-Cultural Adaptation, and Validation of Measurement Instruments: A Practical Guideline for Novice Researchers. J. Multidiscip. Healthc..

