# Supplementary material for: The Italian Version of the Drooling Impact Scale: Translation and Psychometric Validation in Children with Neurodevelopmental Conditions
Source: Children (Basel). 2026 May 29;13(6):757. doi: 10.3390/children13060757 (PMC13297747; doi:10.3390/children13060757)
Supplement: Supplementary file 1 [file children-13-00757-s001.zip › children-4314755-supplementary.pdf]

Supplementary material. File S1: Translation process of the Italian version of the DIS

**Original Item**	**Translation 1**	**Translation 2**	**Reconciled Version**	**Back-Translation**	**Final Version after caregivers feedback**
How frequently did your child dribble?	Quanto frequentemente suo/a figlio/a ha avuto scialorrea?	Con quale frequenza suo figlio ha sbavato?	Con quale frequenza suo figlio ha sbavato	How often has your child dribbled?	Con quale frequenza suo/a figlio/a ha perso saliva?
How severe was the drooling?	Quanto severa era la scialorrea?	Quanto è stata grave la salivazione?	Quanto è stata abbondante la perdita di saliva?	How abundant was the saliva loss?	Quanto è stata abbondante la perdita di saliva?
How many times a day did you have to change bibs or clothing due to drooling?	Quante volte al giorno ha dovuto cambiare bavaglini o indumenti a causa della scialorrea?	Quante volte al giorno hai dovuto cambiare i bavaglini o i vestiti a causa della saliva?	Quante volte al giorno hai dovuto cambiare i bavaglini o i vestiti a causa della saliva?	How many times a day did you have to change bibs or clothes because of drooling?	Quante volte al giorno ha dovuto cambiare bavaglini o vestiti a causa della perdita di saliva?
How offensive was the smell of the saliva on your child?	Quanto era sgradevole l'odore della saliva su suo/a figlio/a?	Quanto è stato sgradevole l'odore della saliva sul tuo bambino?	Quanto era sgradevole l'odore della salvia di suo figlio/a?	How unpleasant was the smell of your child's saliva?	Quanto era sgradevole l'odore della saliva di suo/a figlio/a?
How much skin irritation has your child had due to drooling?	Quanta irritazione della pelle ha avuto suo/a figlio/a a causa della scialorrea?	Quanta irritazione della pelle ha avuto il tuo bambino a causa della salivazione?	Quanta irritazione della pelle ha avuto suo/a figlio/a a causa della salivazione?	How much skin irritation has your child had because of the drooling?	Quanta irritazione della pelle ha avuto suo/a figlio/a a causa della salivazione?
How frequently did your child's mouth need wiping?	Con quale frequenza ha avuto bisogno di asciugare la bocca di suo/a figlio/a?	Con quale frequenza è stato necessario pulire la bocca?	Con quale frequenza è stato necessario asciugare la bocca suo/a figlio/a?	How often was it necessary to wipe your child's mouth?	Con quale frequenza ha dovuto asciugare la bocca di suo/a figlio/a?
How embarrassed did your child seem to be about his/her dribbling?	Quanto sembrava imbarazzato/a suo/a figlio/a a causa della scialorrea?	Quanto il tuo bambino sembrava imbarazzato dalla sua salivazione?	Quanto sembrava imbarazzato/a suo/a figlio/a a causa della salivazione?	How embarrassed did your child seem to be because of drooling?	Quanto sembrava imbarazzato/a suo/a figlio/a per la perdita di saliva?

How much do you have to wipe or clean saliva from household items, e.g. toys, furniture, computers?	Quanto ha dovuto pulire la saliva dagli oggetti domestici, giocattoli, mobili, computer, ecc.?	Quanto spesso è stato necessario pulire o rimuovere la saliva da oggetti domestici, come giocattoli, mobili, computer, ecc.?	Quanto spesso è stato necessario pulire o rimuovere la saliva da oggetti domestici, come giocattoli, mobili, computer, ecc.?	How often was it necessary to clean or remove saliva from household objects, such as toys, furniture, computers, etc.?	Quanto spesso ha dovuto pulire o asciugare la saliva da oggetti della casa (giochi, mobili, computer, ecc.)?
To what extent did your child's drooling affect his or her life?	In che misura la scialorrea di suo/a figlio/a ha influenzato la sua vita?	In che misura la salivazione del tuo bambino ha influenzato la sua vita?	In che misura la salivazione del tuo bambino ha influenzato la sua vita?	o what extent has your child's salivation affected his or her life?	In che misura la perdita di saliva ha influenzato la vita di suo/a figlio/a?
To what extent did your child's dribbling affect you and your family's life?	In che misura la scialorrea di suo/a figlio/a ha influenzato la sua vita e quella della tua famiglia?	In che misura la salivazione del tuo bambino ha influenzato la tua vita e quella della tua famiglia?	In che misura la salivazione del tuo bambino ha influenzato la tua vita e quella della tua famiglia?	To what extent has your child's salivation affected your life and that of your family?	In che misura la perdita di saliva di suo/a figlio/a ha influenzato *la sua vita personale e quella della sua famiglia*?
